# Dynamic changes in mental health status related to the COVID-19 pandemic among health care workers and inpatients in China

**DOI:** 10.3389/fpsyt.2022.956068

**Published:** 2022-10-03

**Authors:** Yujun Tong, Qian Zhang, Xiaoran Wang, Yanlin Du, Dong Chang, Yong Cui, Xinchun Duan

**Affiliations:** ^1^Department of Thoracic Surgery, Beijing Friendship Hospital, Capital Medical University, Beijing, China; ^2^Clinical Epidemiology and EBM Unit, National Clinical Research Center for Digestive Diseases, Beijing Friendship Hospital, Capital Medical University, Beijing, China

**Keywords:** COVID-19, anxiety, depression, healthcare workers, mental health

## Abstract

**Background:**

Exposure to coronavirus disease 2019 (COVID-19) can cause severe mental health problems, the dynamics of which remain unclear. This study evaluated the mental status of frontline health care workers (FHWs) and suspected infected patients (SIPs) during different periods of the COVID-19 outbreak.

**Materials and methods:**

Demographic and psychological data were collected through a cross-sectional survey of 409 participants in a hospital from 20 January to 7 August 2020. COVID-19 outbreaks were divided into three periods owing to the time, place, and scale, including the national outbreak period (a nationwide pandemic period from 20 January to 8 April 2020), sporadic period (a stable period from 9 April to 10 June), and local epidemic period (a local pandemic in Beijing from 11 June to 7 August 2020). Acute psychological disorders (APDs), including symptoms of anxiety and depression, were assessed using the Zung Self-Rating Anxiety/Depression Scale (SAS/SDS).

**Results:**

A total of 206 FHWs and 203 SIPs completed the electronic questionnaire. Overall, the prevalence rates of anxiety and depression among SIPs were 3.9 and 19.4%, respectively, while significantly higher prevalence rates (17.7 and 25.1%) were found among FHWs, *P*-value < 0.05. Psychological status among SIPs did not differ significantly across the three periods. The FHWs were more vulnerable, as their SAS and SDS scores and almost all the dimension scores were significantly higher during the local epidemic period than during the national outbreak and sporadic periods (all *P*-values < 0.001). The prevalence of anxiety (34.41%) and depression (41.94%) was significantly higher during the local epidemic period (*P* < 0.001). Logistic and linear mixed models showed that age, sex, and doctor-patient ratio especially, independently influenced most dimension scores of SAS and SDS among FHWs (*P* < 0.05).

**Conclusion:**

Compared to the COVID-19 epidemic at the national level, the local epidemic had a greater influence on FHWs’ mental health. More attention should be given to the workload of FHWs.

## Introduction

Coronavirus disease 2019 (COVID-19) currently represents an unprecedented threat to human health worldwide (1, 2). The first cases of this novel coronavirus (3, 4) were reported in Wuhan, the capital city of Hubei Province, China, in December 2019. Subsequently, the virus was identified as severe acute respiratory syndrome coronavirus 2 (SARS-CoV-2) by the World Health Organization. The disease has a very strong infectious ability, such that it rapidly spread within a few months and finally became a worldwide health threat. The World Health Organization announced that as of 30 March 2021, 126,372,442 cases had been diagnosed, and 2,769,696 persons had died from COVID-19 due to the high mortality rate and lack of effective treatment (5). The primary routes of transmission include short-distance person-to-person contact, respiratory droplets, and aerosols. The pandemic has relentlessly affected normal social order, caused social panic, and seriously influenced public mental health (6), especially for those frequently exposed to high-risk environments.

Frontline health care workers (FHWs) are the most important force in preventing the spread of COVID-19 and protecting public health (7). However, due to factors such as direct exposure to infectious individuals, a shortage of protective equipment, prolonged separation from family and friends, and even stigmatization (8), FHWs often experience acute psychological disorders (APDs), such as anxiety and depression (9–11). As early as May 2020, the International Council of Nurses reported that more than 90,000 medical workers had been infected with COVID-19. This figure is likely to be conservative because countries were busy combatting the pandemic. Studies (12, 13) have shown that during the outbreak of SARS and Middle East respiratory syndrome (MERS), many frontline staff were infected in their workplaces, which often provoked their loss of emotional control and finally the emergence of APDs.

The effective control of the COVID-19 outbreak in Wuhan was followed by a short period of tranquility. However, the calm soon ended in June 2020 with a local outbreak in the Xinfadi Agricultural Wholesale Market in Fengtai District, Beijing. At this time, increasing numbers of medical staff devoted themselves to handling the drastically increasing number of suspected infected patients (SIPs). The state of the spread of COVID-19 worldwide is not positive. Such a serious situation requires not only effective treatment programs but also more medical staff on the frontlines to combat the pandemic. Although doctor-patient mental health is a concern among scholars, the periods covered in most studies are relatively short (3, 14). Therefore, this study investigated the dynamic changes in APDs caused by the COVID-19 pandemic among health care workers and inpatients over a relatively long period, with the main purpose of providing important evidence for psychological interventions.

## Materials and methods

### Study design, participants, and data collection

**FIGURE 1:**
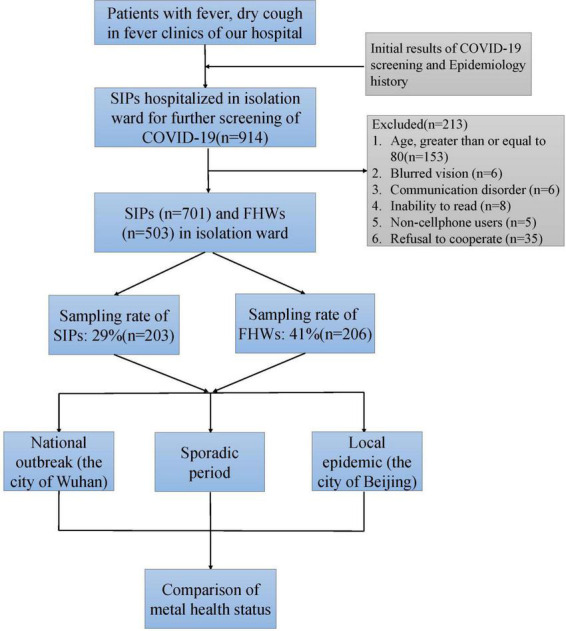
Consolidated Standards of Reporting Trials (CONSORT) diagram of the study. *COVID-19*, coronavirus disease 2019; *FHWs*, frontline health care workers; *SIPs*, suspected infected patients.

This study was a cross-sectional survey conducted from 20 January to 7 August 2020, to observe the different psychosocial status of FHWs and SIPs during outbreaks of COVID-19 (Figure 1). During this time, a total of 503 FHWs were sent to isolation wards to combat the COVID-19 epidemic, each of whom worked in a totally closed environment for 3 weeks. According to the COVID-19 prevention policy, 914 consecutive SIPs were hospitalized in the isolation ward for a definitive diagnosis. A total of 41% of FHWs and 29% of SIPs in our hospital during this period were sampled for the psychological health status survey. All the data were collected by questionnaire on a professional online assessment platform.^1^ Participants completed anonymous self-evaluation forms in the Mini Program provided by WeChat APP linked to the survey website using their own cell phones with each patient submitting questionnaire only once. The exclusion criteria included old age (80 years or above), blurred vision, communication disorders, illiteracy, non-use of cell phones, or refusal to cooperate. All participants were enrolled from isolation wards in Beijing Friendship Hospital, which is a third-level grade A general hospital in Beijing affiliated with Capital Medical University. During the COVID-19 outbreak period, the isolation wards mainly received patients with suspected infection from fever clinics. SIPs were screened by throat swab nucleic acid tests at least twice with an interval of 24 h. The results were reviewed by the team of chief examiners. If COVID-19 infection was confirmed, the infected patients were transferred to a designated hospital for further treatment. The other patients were released from the isolation wards. All the enrolled participants signed informed consent forms, and the study was approved by the Ethics Committee of Beijing Friendship Hospital (2020-P2-161-01).

**FIGURE 2:**
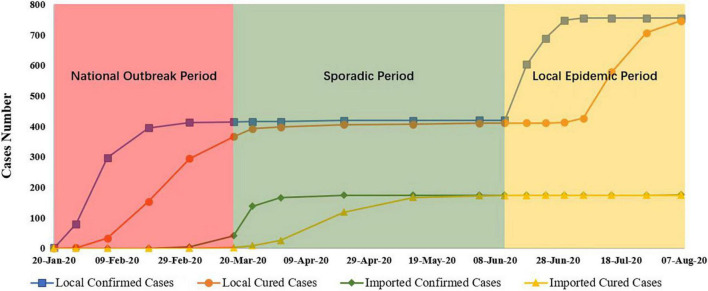
COVID-19 cases during the national outbreak, sporadic, and local epidemic periods in Beijing. Data were obtained from the Beijing Municipal Health Commission (available at http://wjw.beijing.gov.cn/). *COVID-19*, coronavirus disease 2019.

The demographic characteristics included age, sex, area of residence (Fengtai District or other districts), education status (high school or below, bachelor’s degree, master’s degree, and doctoral degree), marital status (single, married, and divorced), and number of children in the participant’s family (no children, one or more children). Participants were classified into three groups according to the enrolment date: national outbreak period, sporadic period and local epidemic period (Figure 2). The COVID-19 outbreak in Wuhan lasted from 20 January to 8 April 2020, and the number of local infected cases in Beijing increased during this period, which was defined as the national outbreak period. From 9 April to 10 June 2020, the epidemic in Wuhan was effectively controlled, and the number of local confirmed cases in Beijing remained stable, without a remarkable increase. This period was defined as the sporadic period. From 11 June to 7 August 2020, a new COVID-19 outbreak occurred in the Xinfadi Agricultural Wholesale Market in Fengtai District, Beijing, and the number of cases increased rapidly. This period was called the local epidemic period.

### Assessment criteria

The symptoms of anxiety and depression were assessed with the Zung Self-Rating Anxiety/Depression Scale (SAS/SDS) (15, 16). These scales have been widely applied in clinical practice and scientific research, showing good reliability and convincing results in the Chinese population (17–19). The SAS and the SDS both include 20 items, with each item scored on a scale from 1 to 4, indicating none or a little of the time, some of the time, a good part of the time, and most or all of the time. The participants responded according to their psychological and physical symptoms within the past week. According to convention in China, the threshold score of anxiety on the self-reported scale is 50. Scores in the range of 50–59 indicate slight anxiety, scores from 60 to 69 indicate moderate anxiety, and scores above 70 indicate severe anxiety. For depression, the cut-off score is 53, with scores in the range of 53–62 implying slight depression, scores ranging from 63 to 72 indicating moderate depression, and scores above 73 indicating severe depression.

Physical symptoms of anxiety and depression can manifest in multiple systems, such as the cardiovascular, digestive, respiratory, skeletal and muscle, urinary, and reproductive systems. The related physical complaints are often composed of factors in several dimensions. The SAS/SDS includes only the abovementioned physical symptoms. Therefore, the SAS/SDS is an appropriate instrument for dimensional analysis and theoretically supports the detection of the cause of mental disease.

A four-factor structure of the SAS/SDS (20, 21), which is generally stable and significantly correlated with relevant variables, was adopted in this study. The SAS contained four factors: anxiety and panic (items 1, 2, 3, 4, 18), somatic control (items 5, 9, 13, 17, 19), vestibular sensations (items 6, 10, 11, 12, 14), and gastrointestinal/muscular sensations (items 7, 8, 15, 16, 20). The SDS also consisted of four dimensions: core depression (items 1, 3, 6, 14, 17, 18, 19, 20), cognitive depression (items 10, 11, 12, 16), anxiety (items 4, 13, 15), and somatic control (items 5, 7, 9). Items 2 and 8 were not included.

### Statistical analysis

The independent continuous variables, including age, SAS score, SDS score and dimension score, were described as means with standard deviations (SDs) and analyzed by ANOVA tests among the three-period groups. Categorical variables, including sex, area of residence, education status, marital status, and the number of children, were described as frequencies (%) and analyzed by the chi-square test or Fisher’s exact test. Bonferroni-adjusted tests were used for multiple comparisons. Univariate and stepwise multivariate logistic regression models were used to calculate the factors influencing anxiety and depression status. All the collected characteristics were entered into the multivariate logistic regression models, and only significant variables remained in the final models. Odds ratios and 95% CIs were calculated for each variable. Linear mixed models were used to calculate the influencing factors with the SAS score, SDS score and dimension score. SAS statistical software version 9.4 (SAS Institute Inc.) was used for statistical analysis. All statistical analyses were tested at a significance level of 0.05 (two-sides).

## Results

### Demographic characteristics of the enrolled health care workers and patients

A total of 203 health care workers assigned to isolation wards during the COVID-19 pandemic period from January to August were enrolled and completed the survey. The demographic characteristics of the enrolled FHWs are shown in Table 1. Across the three periods (national outbreak period, sporadic period, and local epidemic period), there was no significant difference among the medical staff assigned to the isolation wards in terms of age, with most staff being approximately 30 years old (*F* = 0.724, *P* = 0.696). The number of female workers was almost double the number of male workers (*P* = 0.801). The FHWs surveyed in the local epidemic period had lower education levels than those surveyed in the previous periods, with 34.41% of FHWs having a master’s degree or higher vs. 54.10 and 57.14% in the national outbreak period and sporadic period, respectively (*P* = 0.011). The FHWs surveyed in different periods showed no significant differences regarding other demographic characteristics, including marital status, number of children in the participant’s family, and area of residence. Notably, the workload differed significantly across the three periods and presented an upward trend. The doctor-patient ratio was only 0.75 during the national outbreak period and rose to 4.21 during the local epidemic period, which meant that medical resources were so limited that one doctor had to treat 4–5 patients in the isolation wards during the local epidemic period. A total of 206 SIPs in the isolation wards were enrolled and completed the survey. As shown in Table 1, the demographic characteristics of patients in the isolation wards were similar among patients surveyed during the national outbreak period, sporadic period and local epidemic period (all *P*-values > 0.05).

**TABLE 1:** The demographic characteristics of enrolled health care workers and patients during the national outbreak period, sporadic period and local epidemic period.

	National outbreak period	Sporadic period	Local epidemic period	F/χ^2^ value	*P*-value
FHWs	*n* = 61	*n* = 49	*n* = 93		
Age	31.30 ± 4.41	30.78 ± 4.24	31.83 ± 5.79	0.724	0.696
**Sex**					
Male	20 (32.79)	14 (28.57)	26 (27.96)	0.443	0.801
Female	41 (67.21)	35 (71.43)	67 (72.04)		
**Area of residence**					
Fengtai district	10 (16.39)	4 (8.16)	14 (15.05)	1.777	0.411
Other district	51 (83.61)	45 (91.84)	79 (84.95)		
**Education level**					
Bachelor’s or below	28 (45.90)	21 (42.86)	61 (65.59)	9.093	0.011
Master’s or above	33 (54.10)	28 (57.14)	32 (34.41)		
**Marital status**					
Not married	21 (34.43)	16 (32.65)	31 (33.33)	0.040	0.980
Married or divorced	40 (65.57)	33 (67.35)	62 (66.67)		
**Children**					
None	33 (54.10)	29 (59.18)	40 (43.01)	3.875	0.144
1 or more	28 (45.90)	20 (40.82)	53 (56.99)		
Doctor-patient ratio	0.75	1.37	4.21	202	<0.001
Patients	*n* = 33	*n* = 35	*n* = 138		
Age	38.09 ± 11.80	37.46 ± 12.00	39.92 ± 14.00	0.446	0.800
**Sex**					
Male	14 (42.42)	20 (57.14)	73 (52.90)	1.627	0.443
Female	19 (57.58)	15 (42.86)	65 (47.10)		
**Area of residence**					
Fengtai district	10 (30.30)	14 (40.00)	43 (31.16)	1.803	0.582
Other district	23 (69.70)	21 (60.00)	95 (68.84)		
**Education level**					
Bachelor’s or below	29 (87.88)	27 (77.14)	127 (92.03)	#	0.060
Master’s or above	4 (12.12)	8 (22.86)	11 (7.97)		
**Marital status**					
Not married	12 (36.36)	10 (28.57)	39 (28.26)	0.861	0.650
Married or divorced	21 (63.64)	25 (71.43)	99 (71.74)		
**Children**					
None	15 (45.45)	11 (31.43)	45 (32.61)	2.118	0.347
1 or more	18 (54.55)	24 (68.57)	93 (67.39)		

#Fisher’s exact test. FHWs, Health care workers.

### The psychological status of participants in the isolation ward during the COVID-19 pandemic

**FIGURE 3:**
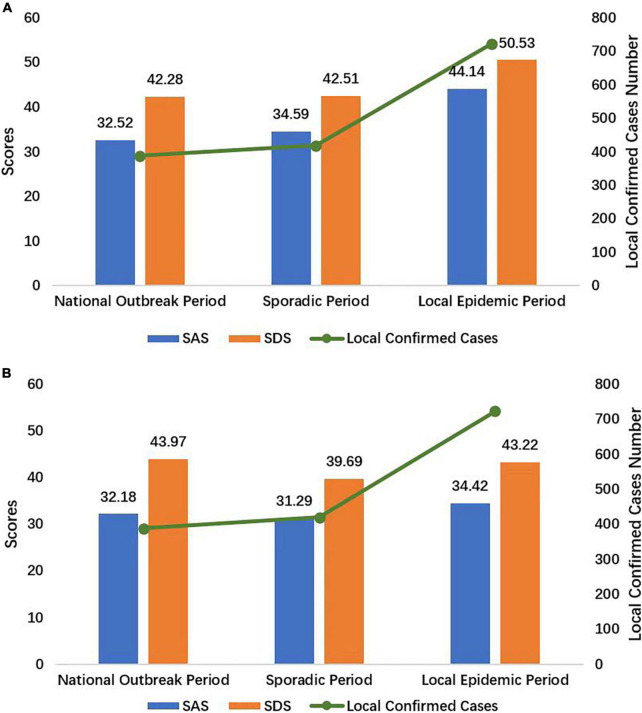
The SAS and SDS scores of FHWs **(A)** and SIPs **(B)** during the national outbreak, sporadic and local epidemic periods. *SAS*, the Zung Self-Rating Anxiety Scale; *SDS*, the Zung Self-Rating Depression Scale; FHWs, frontline health care workers; SIPs, suspected infected patients.

The psychological status of health care workers in the isolation wards was significantly poor during the local epidemic period (Table 2). The SAS and SDS scores and the scores for almost all the dimensions of the SAS/SDS, except for the second dimension of the SDS (*P* = 0.09), were higher in the local epidemic period than in the national outbreak and sporadic periods (all *P*-values < 0.001). The health care workers assigned to isolation wards during the local epidemic period had SAS and SDS scores of 44.14 ± 14.32 and 50.53 ± 13.36, respectively, which were 8–10 points higher than the scores in the national outbreak and sporadic periods (Figure 3A). Furthermore, the proportion of health care workers with anxiety (34.41%) and depression (41.94%) was higher during the local epidemic period (*P* < 0.001). The prevalence of anxiety and depression in FHWs during the national outbreak period and sporadic period was 1.6 and 13.1% and 6.1 and 8.1%, respectively. The overall incidence of anxiety and depression in FHWs was 17.7 and 25.1%, respectively.

**TABLE 2:** Depression and anxiety status and scores for the enrolled health care workers and patients.

	National outbreak period	Sporadic period	Local epidemic period	F/χ^2^ value	*P*-value
FHWs	*n* = 61	*n* = 49	*n* = 93		
SAS scores	32.52 ± 6.08	34.59 ± 7.62	44.14 ± 14.32*	34.211	<0.001
1	60 (98.36)	46 (93.88)	61 (65.59)	33.084	<0.001
≥2	1 (1.64)	3 (6.12)	32 (34.41)		
F1	7.73 ± 1.75	8.06 ± 2.09	10.47 ± 4.29*	19.736	<0.001
F2	9.04 ± 2.87	9.49 ± 2.89	14.35 ± 4.90*	57.31	<0.001
F3	6.84 ± 0.98	7.53 ± 1.87	8.53 ± 3.08**	15.507	<0.001
F4	8.61 ± 2.36	9.13 ± 2.71	10.42 ± 3.88**	8.846	0.012
SDS scores	42.28 ± 10.06	42.51 ± 9.01	50.53 ± 13.36*	20.851	<0.001
1	53 (86.89)	45 (91.84)	54 (58.06)	26.142	<0.001
≥2	8 (13.11)	4 (8.16)	39 (41.94)		
D1	15.41 ± 4.80	15.48 ± 4.27	18.21 ± 5.62*	13.289	0.001
D2	10.64 ± 2.72	10.28 ± 2.86	11.37 ± 3.37	4.812	0.09
D3	5.29 ± 1.93	5.43 ± 1.68	7.06 ± 2.87*	19.391	<0.001
D4	5.49 ± 1.86	5.48 ± 1.80	7.50 ± 2.86*	27.354	<0.001
D5	5.10 ± 1.55	5.43 ± 1.26	6.01 ± 1.80**	11.648	0.003
SIPs	*n* = 33	*n* = 35	*n* = 138		
SAS scores	32.18 ± 7.18	31.29 ± 6.79	34.42 ± 8.83	4.969	0.083
1	33 (100.00)	33 (94.29)	132 (95.65)	#	0.490
≥2	0	2 (5.71)	6 (4.35)		
F1	7.73 ± 1.75	7.21 ± 1.69	8.36 ± 2.77	5.797	0.055
F2	9.85 ± 3.81	9.57 ± 3.05	10.73 ± 3.99	2.77	0.25
F3	6.63 ± 0.80	6.71 ± 1.64	7.03 ± 1.66	5.1	0.078
F4	7.65 ± 1.92	7.43 ± 1.71	7.93 ± 2.41	0.555	0.758
SDS scores	43.97 ± 10.80	39.69 ± 8.41	43.22 ± 11.39	2.611	0.271
1	26 (78.79)	32 (91.43)	108 (78.26)	3.175	0.205
≥2	7 (21.21)	3 (8.57)	30 (21.74)		
D1	17.20 ± 6.21	15.07 ± 3.59	16.98 ± 5.23	2.244	0.326
D2	10.30 ± 3.42	9.32 ± 3.57	9.69 ± 3.72	1.454	0.483
D3	5.76 ± 2.02	5.32 ± 2.21	5.59 ± 2.19	1.406	0.495
D4	4.85 ± 1.55	4.75 ± 1.24	5.54 ± 1.95	6.466	0.039
D5	5.49 ± 1.17	4.79 ± 1.41	5.01 ± 1.62	4.574	0.102

#Fisher’s exact test. *The variables in the local epidemic period were significantly different from those in both the national outbreak period and the sporadic period. **The variables in the local epidemic period were significantly different from those in the national outbreak period. SAS: F1, anxiety and panic (items 1, 2, 3, 4, 18); F2, somatic control (items 5, 9, 13, 17, 19); F3, vestibular sensations (items 6, 10, 11, 12, 14); F4, gastrointestinal/muscular sensations (items 7, 8, 15, 16, 20). SDS: D1, core depressive factor (items 1, 3, 6, 14, 17, 18, 19, 20); D2, cognitive factor (items 10, 11, 12, 16); D3, anxiety factor (items 4, 13, 15); D4, somatic factor (items 5, 7, 9); D5, (items 2, 8).

The psychological status of the patients in the isolation wards was stable overall (Figure 3B) and showed no significant differences across the three periods (Table 2). The prevalence of anxiety and depression among the SIPs was 0 and 21% during the national outbreak period, 5.7 and 8.6% during the sporadic period, and 4.4 and 21.7% during the local epidemic period, respectively. The average incidence of anxiety and depression among the SIPs was 3.9 and 19.4%, respectively.

**FIGURE 4:**
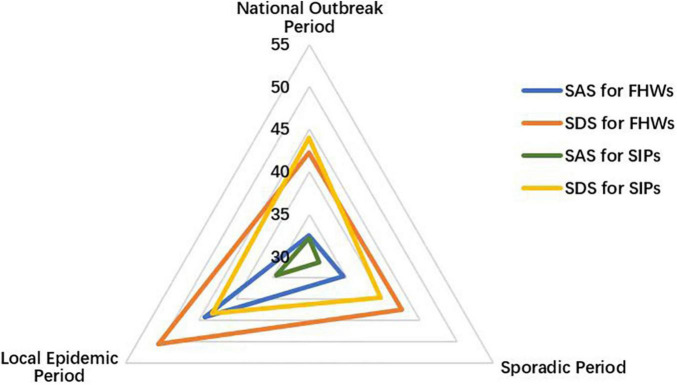
The SAS and SDS scores for frontline health care workers (FHWs) and suspected infected patients (SIPs). *SAS*, the Zung Self-Rating Anxiety Scale; *SDS*, the Zung Self-Rating Depression Scale.

There were evident changes in health care workers’ and patients’ psychological status across the three periods (Figure 4). A comparison of the SAS and SDS scores of the patients in the isolation wards showed an approximately equilateral triangle indicating similar values in each period. In contrast to the patients, the health care workers in the isolation wards scored much higher during the local epidemic period, with skewness in the triangle.

### Comparison of the Zung self-rating anxiety scale and the Zung self-rating depression scale scores between frontline health care workers and suspected infected patients during the periods

**FIGURE 5:**
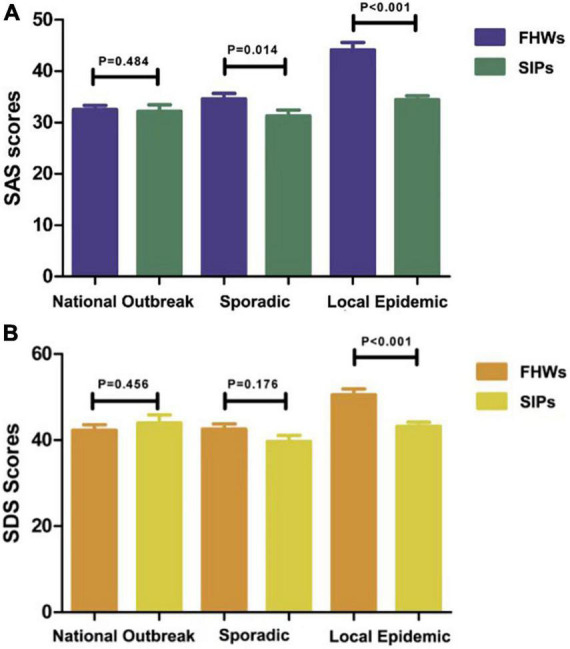
Comparison of SAS **(A)** and SDS **(B)** scores of frontline health care workers (FHWs) and suspected infected patients (SIPs) during different periods.

Figure 5 and Table 2 show the differences between the scores of FHWs and SIPs during the COVID-19 pandemic. There was no significant difference between FHWs and SIPs during the national outbreak period (*P* = 0.484 and *P* = 0.456). In the sporadic period, the SAS scores of the FHWs (34.59 ± 7.62) were higher than those of the SIPs (31.29 ± 6.79), with a *p*-value of 0.014. There was no significant difference in SDS scores between FHWs and SIPs during the sporadic period (*P* = 0.176). In the local epidemic period, the SAS and SDS scores of the FHWs were both higher than those of the SIPs (*P* < 0.001).

The anxiety rate in FHWs was 17.7%, which was higher than that in SIPs (3.9%) (χ^2^ = 20.430, *P* < 0.001). The depression rates in SIPs and FHWs were 19.4 and 25.1%, respectively (χ^2^ = 1.924, *P* = 0.165).

### Factors influencing anxiety and depression among doctors in isolation wards during the COVID-19 pandemic

The multivariate logistic regression models (Table 3) showed that age (*OR* = 0.248, 95% CI: 0.068–0.905) and the doctor-patient ratio (*OR* = 2.434, 95% CI: 1.705–3.476) were independent factors of anxiety, while for depression, only the doctor–patient ratio was an independent factor (*OR* = 1.718, 95% CI: 1.369–2.156).

**TABLE 3:** Factors influencing anxiety and depression status in univariate and multivariate logistic regression models.

	Univariate logistic regression model			Multivariate logistic regression model		
		
	OR	95% CI	*P*	OR	95% CI	*P*
**Anxiety**						
Age	0.384	0.111–1.330	0.131	0.248	0.068–0.905	0.035
**Sex**						
Male	Ref	Ref	Ref			
Female	2.389	0.938–6.084	0.068			
**Area of residence**						
Fengtai district	Ref	Ref	Ref			
Other district	0.99	0.349–2.807	0.985			
**Education level**						
Bachelor’s degree or below	Ref	Ref	Ref			
Master’s degree or above	0.531	0.249–1.131	0.101			
**Marital status**						
Not married	Ref	Ref	Ref			
Married or divorced	0.751	0.357–1.581	0.451			
**Children**						
None	Ref	Ref	Ref			
1 or more	1.012	0.492–2.080	0.974			
Doctor-patient ratio	2.338	1.644–3.326	<0.001	2.434	1.705–3.476	<0.001
**Depression**						
Age	0.287–1.728	0.444				
**Sex**						
Male	Ref	Ref	Ref			
Female	1.009	0.503–2.024	0.979			
**Area of residence**						
Fengtai district	Ref	Ref	Ref			
Other district	0.814	0.335–1.982	0.651			
**Education level**						
Bachelor’s degree or below	Ref	Ref	Ref			
Master’s degree or above	0.962	0.509–1.820	0.906			
**Marital status**						
Not married	Ref	Ref	Ref			
Married or divorced	1.285	0.645–2.558	0.476			
**Children**						
None	Ref	Ref	Ref			
1 or more	1.63	0.857–3.098	0.136			
Doctor-patient ratio	1.718	1.369–2.156	<0.001	1.718	1.369–2.156	<0.001

OR: odds ratio.

The linear mixed models (Table 4) showed that age influenced the total SAS (*P* = 0.011), total SDS (*P* = 0.029), F1 (*P* = 0.007), F2 (*P* = 0.011), F3 (*P* = 0.026), D1 (*P* = 0.038), and D4 (*P* = 0.014) scores. Sex also independently influenced the total SAS (*P* = 0.030), F2 (*P* = 0.037), F3 (*P* = 0.040), and F4 (*P* = 0.049) scores. The doctor-patient ratio independently influenced most of the dimension scores, including total SAS and SDS, F1, F2, F3, F4, D1, D3, D4, and D5 scores (all *P* < 0.005).

**TABLE 4:** Influencing factors in SAS scores, SDS scores, and dimension scores in the linear mixed model.

		Mixed model	
	
		β	*P*
SAS	Age	−0.475	0.011
	Sex	4.129	0.030
	Doctor-patient ratio	3.480	<0.001
F1	Age	−0.149	0.007
	Doctor-patient ratio	0.858	<0.001
F2	Age	−0.172	0.011
	Sex	1.434	0.037
	Doctor-patient ratio	1.615	<0.001
F3	Age	−0.090	0.026
	Sex	0.844	0.040
	Doctor-patient ratio	0.469	<0.001
F4	Sex	1.123	0.049
	Doctor-patient ratio	0.527	<0.001
SDS	Age	−0.436	0.029
	Doctor-patient ratio	2.732	<0.001
D1	Age	−0.182	0.038
	Education level	1.718	0.043
	Doctor-patient ratio	1.014	<0.001
D2	NA	NA	NA
D3	Doctor-patient ratio	0.546	<0.001
D4	Age	−0.100	0.014
	Doctor-patient ratio	0.631	<0.001
D5	Area of residence	−0.740	0.025
	Doctor-patient ratio	0.236	0.001

SAS: F1, anxiety and panic (items 1, 2, 3, 4, 18); F2, somatic control (items 5, 9, 13, 17, 19); F3, vestibular sensations (items 6, 10, 11, 12, 14); F4, gastrointestinal/muscular sensations (items 7, 8, 15, 16, 20). SDS: D1, core depressive factor (items 1, 3, 6, 14, 17, 18, 19, 20); D2, cognitive factor (items 10, 11, 12, 16); D3, anxiety factor (items 4, 13, 15); D4, somatic factor (items 5, 7, 9); D5, (items 2, 8); NA, not applicable.

## Discussion

A total of 409 participants were included in this study, including FHWs and patients from the isolation wards of Beijing Friendship Hospital in Beijing, China. Overall, the incidence of anxiety and depression among FHWs was 17.7 and 25.1%, respectively, which was significantly higher than the incidence among SIPs (3.9 and 19.4%). Furthermore, the occurrence of APDs in patients was basically stable during the three different periods, but the figures for FHWs fluctuated drastically across periods. The scores in the local epidemic period were significantly higher than those in the previous two periods. In addition, age, sex, and doctor-patient ratio were independent risk factors for APDs. It is worth noting that the doctor-patient ratio was the strongest influencing factor for almost all dimensions of the SAS and SDS (only the cognitive dimension in the SDS was not related).

Theoretically, the closeness of contact with COVID-19 determines the risk of being infected and the degree of APD occurrence (22, 23). A recent study (24) based on 43 investigations showed that anxiety and depression were more frequent in FHWs than in non-FHWs. Previous studies (25) showed that anxiety and depression rates were 20.8 and 29.2%, respectively, in infected patients. Among FHWs, the anxiety and depression rates ranged from 38.5 to 44.6% and 21.7 to 50.4%, respectively (22, 26). In our study, the prevalence was consistent with previous studies. It was reported that the reasons for the higher prevalence of APD among FHWs were sociodemographic factors, current and past medical history, psychological and social factors, and job-related factors (24).

The brain is the central organ of stress adaptation that is responsible for sensing and judging the degree of stress and reacting accordingly physiologically and behaviorally (27). Acute and chronic stress can lead to imbalances in the neural circuits of cognition, anxiety and emotion, which in turn affect the physiology and behavior of the whole body through neuroendocrine, autonomic nerve, immune, and metabolic mediators (27, 28). Therefore, when the experience of tension and danger goes beyond the body’s short-term adaptive capability, neural circuits become blocked, which results in mental disorders, such as schizophrenia, anxiety, and depression (28). This can explain why the occurrence of anxiety, depression and other psychological disorders was significantly higher among FHWs than among SIPs and other groups. FHWs were continuously exposed to health-damaging circumstances (29), while SIPs could be discharged after a short stay in the hospital when they tested negative.

In the present study, we found that the doctor-patient ratio was the strongest risk factor influencing the occurrence of APDs among FHWs by affecting various dimensions of the SAS and SDS, including anxiety and panic, somatic control, vestibular sensations, gastrointestinal/muscular sensation factors, core depression, anxiety, and somatic factors (20, 21). A higher doctor-patient ratio implied that FHWs had to care for and manage more patients. In addition, the high proportion of older individuals among SIPs meant that FHWs wearing protective clothing had to be more careful and perform more communication, medical documentation and complex treatments. Simultaneously, the increasing number of SIPs led to higher working hours and workload, causing FHWs to suffer higher psychological and physical pressures (30–33).

In the early periods of COVID-19, Chinese government and hospitals took effective measures to address mental health problems, for example, adopting the psychological protection measures provided by the International Guidelines for Psychological Crisis Intervention, establishing psychological expert groups in hospitals and creating network mental health consulting services (6). Necessary training regarding professional knowledge, mental health, and protective equipment can build the confidence of health care workers, help them overcome the panic linked to the pandemic, and reduce nosocomial infections, thus reducing the occurrence of APDs (23). Above all, the findings suggest that in the long-term fight against COVID-19, more attention should be given to the workload of FHWs, including the doctor-patient ratio, working hours, and night duty arrangements. In addition, the establishment of critical care isolation wards is particularly important.

One strength of this study is that it tracked the dynamic changes in mental health status among participants in different periods. As a result, it provided objective and reliable results regarding the mental health impacts of the COVID-19 pandemic. A self-rating scale, the Zung Self-Rating Anxiety/Depression Scale (SAS/SDS), was adopted in this study. In addition, an in-depth dimensional analysis was conducted to show the main symptoms of anxiety and depression. However, several limitations exist in this study. First, the mental changes experienced by FHWs before and after isolation could not be followed up on due to the cross-sectional survey design of this study. Second, the convenience sampling methods and limited sample size might lead to selection bias. Third, the SAS and SDS have no diagnostic efficacy, even though they have been used in many psychological studies worldwide.

## Conclusion

The present study reveals that FHWs have a much higher chance of experiencing APDs than do SIPs. Furthermore, the prevalence of anxiety and depression among SIPs remained relatively stable, while the prevalence among FHWs fluctuated drastically, with the highest incidence of anxiety and depression occurring during the local epidemic period. Analysis of the related risk factors proved that age, sex, and especially the doctor–patient ratio were independent risk factors for APDs. Our findings suggest that psychological assistance measures should be implemented not only in the anti-epidemic period but also before and after exposure to COVID-19. In addition, more concern and attention should be given to the workload of FHWs.

## Data availability statement

The raw data supporting the conclusions of this article will be made available by the authors, without undue reservation.

## Ethics statement

The studies involving human participants were reviewed and approved by the Ethics Committee of Beijing Friendship Hospital (2020-P2-161-01). The patients/participants provided their written informed consent to participate in this study.

## Author contributions

YT and QZ collected the questionnaires and clinical data. QZ analyzed the data. YT and XD wrote the manuscript with input from all authors. XD and YC designed the study and reviewed the final manuscript. All authors participated in designing various parts of the study and the interpretation of the results.

## Footnotes

1
www.wjx.cn

## Abbreviations

APDs: acute psychological disorders
COVID-19: coronavirus disease 2019
FHWs: frontline health care workers
MERS: Middle East respiratory syndrome
SARS: severe acute respiratory syndrome
SAS: the Zung Self-Rating Anxiety Scale
SDS: the Zung Self-Rating Depression Scale
SIPs: suspected infected patients.
